# Functional somatic syndromes: asking about exclusionary medical conditions results in decreased prevalence and overlap rates

**DOI:** 10.1186/1471-2458-14-1034

**Published:** 2014-10-04

**Authors:** Susanne Fischer, Urs M Nater

**Affiliations:** Clinical Biopsychology, Department of Psychology, University of Marburg, Gutenbergstrasse 18, 35032 Marburg, Germany

**Keywords:** Diagnostic criteria, Epidemiology, Functional somatic syndromes, Overlap, Population-based, Prevalence

## Abstract

**Background:**

The diagnosis of functional somatic syndromes (FSS) requires 1) presence of somatic symptoms, and 2) absence of medical conditions potentially accounting for these symptoms. Due to the limited feasibility of medical examinations, epidemiological research on FSS has neglected to assess the second criterion. Our objective was therefore to evaluate the implications of considering information on exclusionary medical conditions in epidemiological research on FSS.

**Methods:**

A survey among 3’054 students was conducted. We compared prevalence rates and overlap of 17 FSS obtained by: 1) a *symptom-based strategy* and 2) a *symptom-and-exclusion-based strategy* including information on exclusionary medical conditions.

**Results:**

The *symptom-and-exclusion-based strategy* led to a marked decrease in prevalence rates compared to the *symptom-based strategy*. Furthermore, it resulted in fewer individuals who were affected by multiple FSS.

**Conclusions:**

Adding self-reported information on exclusionary medical conditions leads to a significant decrease in the prevalence and overlap of FSS. More rigorous approaches to studying FSS should be adopted.

## Background

The term ‘functional somatic syndrome’ (FSS) refers to a certain constellation of somatic symptoms that cannot be adequately explained in the context of a known medical condition. Case definitions of the numerous existing FSS therefore each require 1) the presence of at least one characteristic symptom (positive criterion), and 2) the absence of any medical condition that can account for these symptoms (negative criterion). There is a long list of FSS, but among the most prevalent are chronic fatigue syndrome, fibromyalgia syndrome, and irritable bowel syndrome. The diagnostic criteria for FSS are commonly formulated by expert committees; examples are the 1994 Centers for Disease Control and Prevention criteria for chronic fatigue syndrome [1], the Rome III criteria for irritable bowel syndrome (and other functional gastrointestinal disorders) [2], and the 1990 and 2010 American College of Rheumatology criteria for fibromyalgia [3, 4].

These diagnostic criteria are used in clinical practice and research settings, where patients are asked about symptoms (positive criterion), medical records are reviewed, and physical examinations and laboratory tests are performed in order to identify medical conditions considered exclusionary for FSS (negative criterion). This two-step approach, which covers the assessment of both criteria inherent in the definition of FSS, is considered the *gold standard* for diagnosing an FSS. However, epidemiological research is challenged by the limited feasibility of reviewing medical records and/or conducting comprehensive medical examinations, and thus often exclusively relies on self-reported information. Several ways of diagnosing FSS have been adopted to deal with this problem: a) asking patients whether they suffer from a (specific) FSS (*self-reported diagnosis*), b) asking patients whether they have ever received an FSS diagnosis by a physician (*physician diagnosis*), or c) providing patients with a list of characteristic symptoms in accordance with the diagnostic criteria, but without an assessment of exclusionary factors (*symptom-based diagnosis*). Naturally, the approaches leading to these outcomes differ in their ability to cover both the positive and negative criterion of FSS.

It is conceivable from this comparison that the choice of diagnostic strategy may contribute to diverging study findings. In fact, reviews on the epidemiology of each FSS show a broad range of prevalence rates across studies (e.g., [5–7]). Another epidemiological estimate rather specific to research on FSS is the amount of comorbidity among FSS, i.e., the so-called ‘overlap’. With regard to prevalence rates, overlap between FSS has been found to vary substantially [8, 9]. Importantly, studies showing high levels of overlap have led some researchers to propose the existence of only one FSS [10]. These so-called ‘lumpers’ are opposed by other authors, who insist that there are several specific syndromes, and these authors are usually referred to as ‘splitters’ [11]. Thus, the overlap rates can be considered a key parameter in the so-called ‘one vs. many debate’. However, direct evidence on the repercussions of using different diagnostic strategies as a possible reason for the observed discrepancies in prevalence rates and overlap is extremely scarce.

To the best of our knowledge, so far, only one study has directly examined the consequences of using different diagnostic strategies for FSS. In a recent study conducted among female FSS patients and matched controls, Warren and Clauw [12] reported a lack of sensitivity and specificity of *physician diagnoses* (the above-mentioned option b) when compared to *symptom-based diagnoses* (option c). While we fully agree with the authors’ conclusion that ‘queries of symptoms, not diagnoses, are necessary’ (p. 894 in the same article), we believe that merely asking about characteristic symptoms (positive criterion) may result in an overestimation of FSS prevalence (and possibly overlap) rates. In cases in which a thorough medical examination is not feasible (such as in the above-mentioned study designs), we believe it preferable to also obtain self-reported information on medical illnesses considered exclusionary for FSS (negative criterion). In essence, we would argue in favor of a combination of options b) and c) in determining FSS diagnoses in epidemiological studies (*symptom-and-exclusion-based strategy*).

However, the potential impact of this strategy needs to be examined. We aimed to extend the findings reported by Warren and Clauw [12] by comparing two different diagnostic strategies in 17 different FSS in a large, non-clinical sample of young adults. The two strategies were as follows: 1) identifying cases of FSS by means of presenting a list of symptoms that are based on the diagnostic criteria (*symptom-based strategy*), and 2) additionally asking about medical exclusionary criteria (*symptom-and-exclusion-based strategy*). We expected to find 1) a significant decrease in prevalence rates of FSS, and 2) a marked decrease in the extent of overlap between FSS when using the *symptom-and-exclusion-based strategy*. We tested these hypotheses as part of a larger study on the prevalence, overlap, and predictors of FSS [13].

## Methods

### Participants

The recruitment procedure for participants in this study has been described previously elsewhere [13]. In brief, German-speaking students from 23 Swiss colleges and universities were contacted via e-mail through cooperating school administrators, and asked to participate in a web survey on physical and mental well-being. All procedures were in accordance with the ethical standards laid down in the 1964 Declaration of Helsinki, and the web survey study design was approved by the ethics committee of the Canton of Zurich. Written informed consent was obtained from all participants.

### Measurement

We administered a previously developed questionnaire (Questionnaire on Functional Somatic Syndromes; FFSS; [14]). The German version of the FFSS is freely available as a Web supplement to the original article (http://content.karger.com/ProdukteDB/miscArchiv/000/333/298/000333298_sm_supplemental_material.pdf). The FFSS consists of three different parts which are connected via several algorithms. In the first part, a screening section encompassing 52 items on various somatic symptoms was presented. These items represent cardinal symptoms of 17 FSS: Tension-type headache and persistent idiopathic facial pain [15], whiplash-associated disorders (pain of at least 6 months’ duration that is related to an accident), temporomandibular disorders [16], globus and functional chest pain of presumed esophageal origin [17], functional dyspepsia [18], irritable bowel syndrome [2], chronic low back pain (lower back pain of at least 6 months’ duration causing impairment), fibromyalgia syndrome [3], chronic fatigue syndrome [1], multiple chemical sensitivity [19], chronic pelvic pain in men [20] and in women (lower abdominal pain of at least 6 months’ duration), premenstrual syndrome [21] and premenstrual dysphoric disorder [22], and hyperventilation syndrome [23]. The instruction was to rate all current symptoms (‘I suffer from the following complaints:’) according to frequency of occurrence (‘never/rarely’, ‘frequently’, ‘almost always/always’). In addition, the screening part contains dichotomous questions on functional impairment due to symptoms in different areas and a categorical item on the duration of symptoms.

In the second part, if participants reported cardinal symptoms that were at least ‘frequently’ present and characteristic of one of 17 FSS (e.g., abdominal pain in the case of irritable bowel syndrome), additional questions based on diagnostic criteria (e.g., Rome III) were presented. Our questions were based on the most commonly used diagnostic criteria (all publications containing these criteria can be found in the previous section for each FSS). These questions allowed for a detailed understanding of both cardinal and associated symptoms, symptom course and fluctuation, functional impairment, and symptom onset for each FSS. Participants were labelled as having a *‘symptom-based FSS’* if they met the minimum of required positive criteria (e.g., recurrent abdominal pain or discomfort on at least 3 days per month in the last 3 months including changes in bowel movement, with symptom onset at least 6 months previously).

In the third part, those who fulfilled the positive criteria of a specific FSS were subsequently surveyed about health care visits. Importantly, visits related to the previously diagnosed FSS (but not health care visits in general) were of interest at this point (e.g., ‘Have you ever visited a doctor about your abdominal pain/changes in bowel movement?’). Participants who responded with ‘yes’ were ultimately directed to a list of items addressing frequent differential diagnoses (‘What diagnosis did your doctor give you regarding your abdominal pain/changes in bowel movement?’). These lists were again based on the diagnostic criteria for each FSS as cited above. If they reported that no abnormalities had been detected by their doctor that might account for their symptoms (e.g., an inflammatory bowel disease), participants were labelled as having a *‘symptom-and-exclusion-based FSS’*. The FFSS screening part has good psychometric properties regarding both internal consistency (Cronbach’s alpha = 0.94) and retest reliability (r = 0.80 – 0.94).

Prevalence rates and overlap estimations of *symptom-and-exclusion-based FSS* have already been described in our previous report [13].

## Results

### Participants’ characteristics

**Figure 1:**
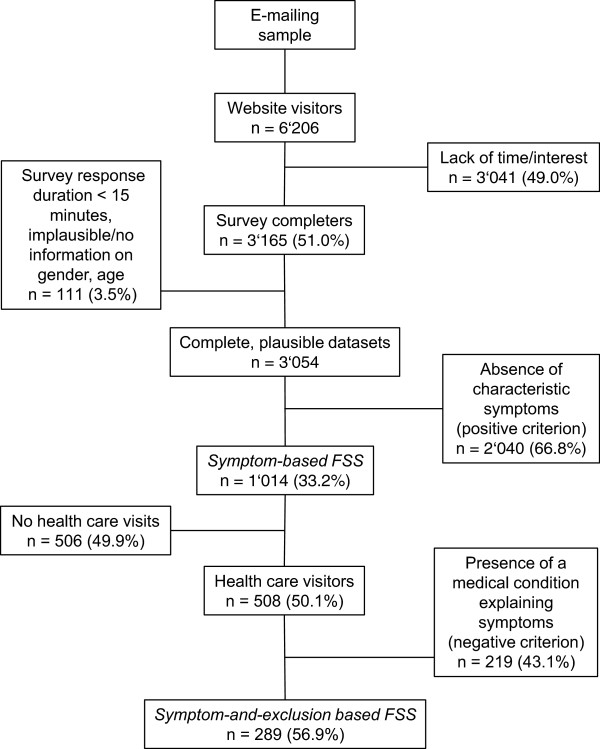
**Recruitment and diagnostic process.**

Our recruitment and data preparation process is visualized in Figure 1. A total number of N = 6’206 participants visited the website and about 51% of them finished the survey. After the exclusion of implausible and incomplete datasets (regarding survey response duration, gender, and age), N = 3’054 datasets remained for further analyses. Out of these 3’054 participants, 2’242 (73.4%) were women and 812 (26.6%) were men. The mean age was 24.6 ± 5.6 (SD) years. Parental household income was almost uniformly distributed across nine predefined categories ranging from less than 3’000 to more than 10’000 Swiss Francs per month (equal intervals across categories).

### Prevalence of FSS

**Figure 2:**
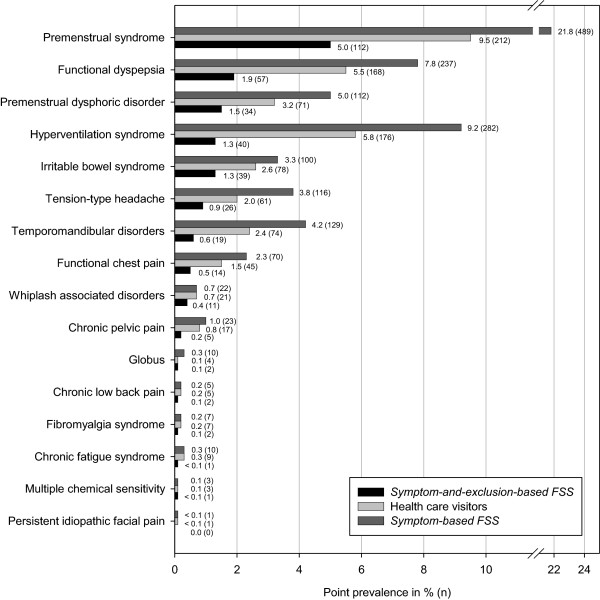
**Prevalence rates of** ***symptom-based FSS*** **, health care visitors, and** ***symptom-and-exclusion-based FSS.***

As illustrated in Figure 1, about one third of our sample endorsed an FSS when using the *symptom-based strategy*. Half of these participants had embarked upon health care visits because of their symptoms. More than half of the health care visitors were not offered a medical explanation for their symptoms and those were thus labelled *symptom-and-exclusion-based FSS* cases. To compare the impact of our two diagnostic strategies on epidemiological data, we calculated the prevalence rates of 17 FSS according to both strategies. We additionally included the health care visitor data for descriptive purposes. No male participant reported suffering from chronic pelvic pain and thus this FSS was excluded from all analyses. The results are illustrated in Figure 2. The prevalence rates of the premenstrual syndrome, premenstrual dysphoric disorder, and chronic pelvic pain all refer to the female population only. In accordance with our first hypothesis, we observed marked decreases in prevalence rates when using a *symptom-and-exclusion-based approach* to diagnosing FSS.

### Overlap between FSS

To evaluate the potential impact of our two diagnostic strategies on the extent of overlap between FSS, we counted the number of FSS per person according to each strategy. The number of *symptom-based FSS* per person ranged from one to eight, with 631 (62.2%) participants reporting only one, 239 (23.6%) reporting two, 92 (9.1%) reporting three, 35 (3.5%) reporting four, 13 (1.3%) reporting five, three (0.2%) reporting six, and one person (0.1%) reporting eight *symptom-based FSS* occurring at the same time. The number of *symptom-and-exclusion-based FSS* ranged from one to four: 227 (78.5%) participants fulfilled criteria for only one, 49 (17.0%) reported two, 12 (4.2%) reported three, and one person (0.3%) reported four *symptom-and-exclusion-based FSS* simultaneously.

We then calculated the number of co-occurring FSS for each strategy separately. Since premenstrual syndrome represents a less severe form of premenstrual dysphoric disorder, the extent of overlap between these syndromes was not evaluated. We first looked at each FSS separately. For instance, within the irritable bowel syndrome group, most people had one additional FSS, but some had up to seven additional syndromes. We did this with every syndrome and computed an average index. Within the *symptom-based FSS* group, 9.2 ± 3.6 different co-occurring syndromes (out of 16) were present on average, whereas individuals with *symptom-and-exclusion-based FSS* fulfilled criteria for an average amount of 3.7 ± 3.0 co-occurring syndromes (out of 15, excluding idiopathic facial pain).

## Discussion

### Summary of study results

In this study, we aimed to evaluate the implications of considering self-reported information on exclusionary medical conditions in epidemiological research on FSS. We compared prevalence rates and overlap of 17 FSS diagnoses obtained by two different diagnostic strategies: a *symptom-based strategy* and a *symptom-and-exclusion-based strategy*. We report two findings that are in accordance with our initial hypotheses: First, the use of medical exclusionary criteria (*symptom-and-exclusion-based strategy*) led to a marked decrease in prevalence rates of FSS when compared to the *symptom-based strategy*. Second, the use of the *symptom-and-exclusion-based strategy* resulted in fewer numbers of individuals who were affected by multiple FSS at the same time. Moreover, it also resulted in fewer overlapping syndromes.

### Integration and interpretation of study results

This is the first study to directly examine the impact of adding information on exclusionary medical conditions on the prevalence of FSS. In a recent report, Warren and Clauw [12] found *symptom-based diagnoses* to be superior to *physician diagnoses* of FSS in terms of sensitivity and specificity. While this is an important finding, with both diagnostic and clinical implications, the results of the present study indicate that a *symptom-based strategy* might, in turn, overestimate prevalence rates of FSS. This is most likely due to a participant’s incorrect attribution of a somatic symptom (e.g., abdominal pain) to a specific FSS (e.g., irritable bowel syndrome), when, in fact, it is part of a medical illness (e.g., Crohn’s disease).

Our finding of a marked decrease in prevalence rates of FSS when considering exclusionary medical conditions is mirrored by other population-based research adopting the *gold standard* procedure, in which patients are first asked about symptoms (positive criterion), followed by physical examinations and laboratory testing (negative criterion). None of these studies explicitly assessed the ramifications of using different diagnostic strategies; however, their detailed reporting of patient screening procedures (e.g., using flow charts) allows the reader to compare the number of participants at each step of this process. For example, before vs. after medical examination by the study investigators, chronic fatigue syndrome was present in 555 vs. 43 individuals in a US-based study [24], and 7.5% vs. 1.6% in a French sample were estimated to have fibromyalgia [25]. Similarly, an in-depth look at the study by Koloski et al. [26], in which an approach comparable to our *symptom-and-exclusion-based strategy* was used in functional gastrointestinal disorders, reveals a more than doubled decrease in prevalence rates before vs. after the exclusion of medical illnesses. This suggests that the use of our *symptom-and-exclusion-based strategy* ‘mimics’ the diagnostic pathway of epidemiological gold standard studies adequately well. For absolute comparisons of prevalence rates obtained by this strategy with findings of other studies, the interested reader is referred to a previously published article by our group [13].

Based on our findings, we further argue that potential misattribution of somatic symptoms to a specific FSS (instead of a medical illness) artificially inflates the extent of overlap between syndromes. Only a small number of population-based studies have examined overlap between multiple (i.e., more than two) FSS [27–30]. On the one hand, two of these studies relied either on *physician-based*[27] or on *symptom-based*[28]*diagnoses*. Their finding of a substantial co-occurrence of FSS is in accordance with our finding of more than nine concomitant syndromes in our *symptom-based FSS* group. Interestingly, in one of these studies, the authors argue that their ‘results support theories suggesting that medically unexplained conditions share a common etiology’ [ [27]; p. 818]. This study, as well as our finding of a considerable overlap between *symptom-based diagnoses*, are therefore in favor of the single syndrome hypothesis [lumpers’ position; [10]]. On the other hand, in another study, a *symptom-and-exclusion-based strategy* was used for the diagnosis of FSS [30]. After re-analyzing their Swedish Twin Registry data, Kato et al. reported that only 2.8% of their participants were characterized by multiple FSS [30]. This percentage is in line with our finding of 4.5% of participants having at least three *symptom-and-exclusion-based FSS*. Based on their findings, the authors conclude that ‘taken together, overlaps among the three functional somatic syndromes were not substantial’ (p. 451). This study, as well as our data obtained by using the *symptom-and-exclusion-based strategy*, thus both lend support to the notion of the existence of multiple specific FSS instead of one single syndrome [splitters’ position; [11]]. Taken together, study findings regarding the overlap between FSS seem to depend heavily on the selected diagnostic strategy, a finding which has important conceptual ramifications (one vs. many debate). Importantly, to answer the question of overlap, and whether FSS are all expressions of the same underlying phenomenon or discrete diagnoses, a different analysis strategy should be employed [see e.g., [31, 32]]. This strategy would ideally combine a factor analysis with latent class analysis. Unfortunately, the hierarchical, modular structure of the herein used FFSS prevented the use of this approach in the current data set.

### Study strengths and limitations

A strength of our study lies in our access to a large, non-clinical sample that was free of any healthcare-seeking bias. Nevertheless, a number of limitations need to be taken into account when interpreting our results. First, the present survey was conducted in a student sample, which cannot be considered representative of the general population. However, as outlined above, our findings are in accordance with general population-based studies, indicating potential generalizability at least to some extent. Second, our strategy of establishing diagnoses of FSS was dependent on health care visits. This led to a reduction of our sample size, and could have potentially resulted in an underestimation of ‘true’ prevalence rates in *symptom-and-exclusion-based FSS*. However, accounting for medical exclusionary conditions is very likely to explain a large proportion of the decrease in prevalence rates, as mirrored by the fact that in 43.1% of cases, a medical explanation for patients’ symptoms was provided by a health care professional. Third, due to the nature of a web-based data collection approach, we were unable to confirm our diagnoses through a physical examination or laboratory assessment in our participants (*gold standard* procedure). This might again have led to an underestimation of ‘true’ prevalence rates in *symptom-and-exclusion-based FSS*, since patients whose symptoms were caused both by an FSS and a medical condition were not counted as FSS cases in this study. In other words, we considered those individuals having a medical condition that explained their symptoms on part as non-cases. Also, some of the exclusionary medical conditions might have been incidental, with the FSS actually causing the symptoms. As illustrated above, our diagnostic strategy does, however, lead to similar decreases in prevalence rates compared to those epidemiological studies using the *gold standard* approach.

## Conclusions

To summarize, we were able to show that including information on exclusionary medical conditions leads to a significant decrease in prevalence and overlap rates of FSS. This may call into question the validity of the findings of a number of epidemiological studies on FSS. In a next step, the validity of our *symptom-and-exclusion-based strategy* should be checked in FSS patients that were diagnosed by the *gold standard procedure*. Also, comparisons of prevalence rates as obtained by our approach with prevalence rates of self-reported and physician diagnoses would be of interest. Future studies should adopt more rigorous approaches to the study of FSS, and combine both the positive and negative criterion inherent in their definition. This is likely to enhance the clinical benefit from epidemiological findings on FSS, with the potential to guide diagnostic and, ultimately, treatment decisions.

## Abbreviations

FSS: Functional somatic syndromes.
